# Hochschulbeschäftigte in der Coronapandemie: Auswirkungen auf die Gesundheit und das Wohlbefinden von Hochschulbeschäftigten mit Kind(ern) im Kita- und Grundschulalter

**DOI:** 10.1007/s11553-021-00898-x

**Published:** 2021-09-15

**Authors:** Kathrin Allgayer, Carolin Bäßler, Regina Jutz, Marlen Niederberger

**Affiliations:** 1 Forschungsmethoden in der Gesundheitsförderung und Prävention, Pädagogische Hochschule Schwäbisch Gmünd, Oberbettringer Str. 200, 73525 Schwäbisch Gmünd, Deutschland; 2 Abteilung Soziologie, Pädagogische Hochschule Schwäbisch Gmünd, Oberbettringer Str. 200, 73525 Schwäbisch Gmünd, Deutschland

**Keywords:** Hochschule, COVID-19, Lebenszufriedenheit, Online-Befragung, Wissenschaftler, University, COVID-19, Life satisfaction, Online survey, Scientist

## Abstract

**Hintergrund:**

Die Coronapandemie erweist sich 2020/21 für die gesamte deutsche Bevölkerung als multidimensionaler Stressfaktor. Erste Studien deuten an, dass diese Zeit insbesondere für berufstätige Eltern mit Kind(ern) herausfordernd ist. Sie sind Belastungsfaktoren ausgesetzt, aus denen sich psychische, soziale und physische Gefährdungen ergeben können.

**Ziel der Arbeit:**

Untersucht werden die Auswirkungen der Coronapandemie auf die Gesundheit und das Wohlbefinden von Hochschulbeschäftigten mit Kind(ern) im Kita- und Grundschulalter aus Sachsen (SN) und Baden-Württemberg (BW).

**Methodik:**

Mittels eines standardisierten Online-Fragebogens wurden Hochschulbeschäftigte in SN und BW zu ihrer Lebens- und Arbeitssituation in der Coronapandemie sowie ihrem subjektiven Wohlbefinden und ihrer Gesundheit befragt.

**Ergebnisse:**

Besonders belastend nehmen die Befragten die Veränderung der sozialen Situation, die Verschlechterung der Balance zwischen Berufs- und Privatleben und das Verschwimmen der Grenzen zwischen Arbeits- und privater Zeit wahr. Die befragten Hochschulbeschäftigten in SN zeigen bei verschiedenen Belastungsfaktoren in Bezug auf die Arbeitssituation signifikant schlechtere Bewertungen als die Befragten in BW. In beiden Bundesländern geben jeweils mehr als die Hälfte der Befragten eher kritische Werte für ihr Wohlbefinden an.

**Schlussfolgerung:**

Die Coronapandemie und die damit einhergehenden Schutzmaßnahmen haben negative Auswirkungen auf das Wohlbefinden von Hochschulbeschäftigten mit Kind(ern) im Kita- und Grundschulalter. Inwieweit sich diese Effekte längerfristig zeigen, wenn beispielsweise strukturelle Maßnahmen im Bereich Homeoffice und Online-Lehre in Hochschulen verstetigt werden, ist zu prüfen.

## Abstract (en)

**Background:**

The coronavirus pandemic turns out to be a multidimensional stressor for the entire German population in 2020/2021. Studies indicate that these corona-related changes are also challenging for university employees with children. Parents were exposed to stress factors, which can be negatively associated with various psychological, social and physical aspects.

**Objectives:**

Investigation of the impact of the coronavirus pandemic on health and well-being of university employees with daycare- and primary school-aged children.

**Methods:**

Using a standardized online questionnaire, university employees in Saxony and Baden-Wuerttemberg were contacted and asked about their living and working situation during the coronavirus pandemic as well as their subjective well-being and health.

**Results:**

The respondents perceive the changes in the social situation, the deterioration of the balance between work and private life and the blurring of boundaries between work and private time as particularly stressful. The respondents in Saxony show significantly worse ratings than in Baden-Wuerttemberg for various stress factors in relation to the work situation. In both states, slightly more than half of the respondents marked categories that indicate a lack of well-being.

**Conclusions:**

The coronavirus pandemic and its accompanying protective measures have had negative effects on the well-being of university employees with children of daycare and elementary school age. The extent to which these effects will become apparent in the longer term, for example, whether structural measures of remote work and online teaching in universities are made permanent, must be examined in the future.

Die Coronapandemie und deren Schutzmaßnahmen bestimmen in den Jahren 2020/21 das öffentliche und private Leben in Deutschland. Durch die Schließungen von Kitas und Schulen sind insbesondere berufstätige Eltern einer Mehrbelastung im Alltag ausgesetzt [8]. Berufliche Rahmenbedingungen können diese abfangen oder verstärken [4]. In der vorliegenden Studie werden die Auswirkungen der Coronapandemie auf die Gesundheit und das Wohlbefinden von Hochschulbeschäftigten mit Kind(ern) im Kita- und Grundschulalter vorgestellt.

## Hintergrund

Die Coronapandemie erweist sich 2020/21 für die gesamte deutsche Bevölkerung als multidimensionaler Stressfaktor, welcher auch negative Auswirkungen auf die Gesundheit derer hat, die nicht an COVID-19 („coronavirus disease 2019“) erkranken [12]. Sie gehen u. a. aus Einschränkungen sozialer Kontakte, geringem Kontrollerleben sowie geringer Selbstwirksamkeitserwartung hervor [2]. Belastungen entstehen auch aufgrund notwendiger Schutzmaßnahmen und Regelungen im Arbeitskontext [13]. Diese Maßnahmen können sekundäre Gefährdungen hervorrufen, was für die Themen Homeoffice/Telearbeit [15] mit zunehmender Entgrenzung von Arbeit und Familie belegt ist [3].

Befunde zeigen, dass coronabedingte Veränderungen insbesondere für berufstätige Eltern herausfordernd sind. Vor allem während der zwei Lockdowns (03.–06.2020 und 12.2020–02.2021), in denen bundesweit Schulen und Kitas geschlossen wurden, waren Eltern einer Mehrbelastung im Alltag ausgesetzt und gezwungen, bspw. durch Homeschooling zusätzliche Aufgaben zu bewältigen [2, 10]. Diese Belastungsfaktoren erzeugen nach bisherigen Befunden verschiedene psychische, soziale und physische Gefährdungen [20]. Eltern mit Kindern unter 6 Jahren erleben in der Coronapandemie verglichen mit Eltern älterer Kinder einen stärkeren Rückgang der allgemeinen Lebenszufriedenheit [8]. Die Situation erscheint für Beschäftigte in nicht-systemrelevanten Berufen belastend, weil sie in den meisten Bundesländern im ersten Lockdown keinen und im zweiten Lockdown nur unter bestimmten Bedingungen einen Anspruch auf eine Notbetreuung hatten [10].

### Corona und Hochschulbeschäftigte

Internationale und nationale Studien belegen Folgen der Coronapandemie auf die Arbeitsproduktivität, die Arbeitsplatzgestaltung und die Gesundheit der Hochschulbeschäftigten [16, 24]. So zeigt eine repräsentative Studie aus Großbritannien, dass 66 % in dieser Zeit ein hohes Stresslevel angeben [25]. Auch an einer Berliner Hochschule geben 71 % der Lehrenden im Sommersemester 2020 an, einer starken allgemeinen Belastung ausgesetzt zu sein [1]. Inwieweit sich diese Folgen insbesondere auf Hochschulbeschäftigte mit Kind(ern) auswirken, wird im vorliegenden Forschungsprojekt untersucht. Dem ganzheitlichen Gesundheitsverständnis der Weltgesundheitsorganisation folgend, werden dabei soziale, physische und psychische Aspekte erhoben [26].

## Methodik

### Studiendesign

Die Studie wurde als quantitative Online-Befragung konzipiert und durch vier Strategien entwickelt:

1. Nutzung etablierter Items zur Messung von Gesundheit und Wohlbefinden: WHO (fünf)-Fragebogen zum Wohlbefinden [17], sozioökonomisches Panel [9], Fragebogen zum aktuellen körperlichen Wohlbefinden [6] sowie zum allgemeinen habituellen Wohlbefinden [27].
2. Sichtung aktueller Studien zur Gesundheit von Beschäftigten in der Coronapandemie,
3. Durchführung fünf qualitativer Expert*inneninterviews aus dem Kontext Hochschule,
4. partizipative Entwicklung des Fragebogens mit Praxispartner*innen aus den Bereichen Gleichstellung, Gesundheitsmanagement und Arbeitsschutz an Hochschulen aus Sachsen (SN) und Baden-Württemberg (BW).

### Stichprobe

Zielgruppe sind Hochschulbeschäftigte mit Kind(ern) im Kita- und Grundschulalter in SN und BW. Die Auswahl der Bundesländer erfolgte aufgrund eines angestrebten Ost-West-Vergleichs, kombiniert mit hohen Inzidenzwerten im Laufe der Coronapandemie 2020. Zudem bildeten BW und SN 2019 die Schlusslichter im Länderranking nach Gleichstellungsaspekten an Hochschulen [14]. Die vergleichsweise niedrigen Anteile an weiblichen Promovierenden und Beschäftigten deuten strukturelle Probleme an, die bereits vor der Coronapandemie existierten, sich aber im Zuge der coronabedingten Herausforderungen als ungünstige Voraussetzung möglicherweise potenzieren.

Die Befragung fand online während des zweiten Lockdowns (11.01.2021 bis 07.02.2021) statt. Die Stichprobe beruht auf einem Convenience-Sampling, d. h. die Hochschulbeschäftigten wurden über Gatekeeper angesprochen. Als Gatekeeper fungierten verschiedene Praxispartner*innen auf der Landesebene aus den Bereichen Gleichstellung und betriebliches Gesundheitsmanagement im Kontext Hochschule, die über E‑Mail-Verteiler der assoziierten Stellen an den Hochschulen verfügten. Zusätzlich wurden E‑Mails an die Rektor*innen aus BW und SN versendet, mit der Bitte zur Verteilung an die Beschäftigten. Auf diese Weise wurden die Beschäftigten über mehrere Kanäle über die Befragung informiert und gebeten, sich zu beteiligen. Allerdings konnte bei dieser Vorgehensweise die Grundgesamtheit der Stichprobe nicht bestimmt und somit die Rücklaufquote nicht berechnet werden.

Insgesamt haben sich 1258 Hochschulbeschäftigte aus BW oder SN an der Befragung beteiligt. Nach einer Bereinigung des Datensatz, bei der nur Befragte berücksichtigt werden, die mindestens 75 % des Fragebogens ausgefüllt haben, verblieben 1104 Teilnehmende. Die Beschreibung der Stichprobe ist in Tab. 1 dargestellt.

**Tab. 1:** Deskriptive Beschreibung der Befragten (Anteile in Klammern; *n* = 1104)

Bundesland	Baden-Württemberg (66 %) *n* = 731	Sachsen (34 %) *n* = 373
*Geschlecht:*		
Männlich	241 (33,0 %)	148 (39,7 %)
Weiblich	488 (66,8 %)	224 (60,1 %)
Divers	2 (0,3 %)	1 (0,3 %)
*Kinderanzahl:*		
Ein Kind	343 (46,9 %)	182 (48,8 %)
Zwei Kinder	325 (44,5 %)	171 (45,8 %)
Drei oder mehr Kinder	63 (8,6 %)	20 (5,4 %)
*Arbeitsverhältnis:*		
Vollzeit	328 (45,4 %)	216 (58,1 %)
Teilzeit	395 (54,6 %)	156 (41,9 %)
Befristet	290 (40,1 %)	210 (56,5 %)
Unbefristet	434 (59,9 %)	162 (43,5 %)
*Beschäftigungsgruppe:*		
Wissenschaft/Lehre	404 (55,5 %)	232 (62,4 %)
Technik/Verwaltung/Wissenschaftsmanagement	321 (44,1 %)	132 (35,5 %)
Sonstiges	3 (0,4 %)	8 (2,2 %)
Mindestens 50 % der Zeit im Home-Office:	588 (83,3 %)	282 (78,6 %)

### Auswertung

Die folgende deskriptive Auswertung stellt die Gesundheit und das Wohlbefinden der Befragten während der Coronapandemie dar. Bei allen Fragen wurden die Befragten gebeten, die Zeit der Coronapandemie von März 2020 bis zum Befragungszeitpunkt zu berücksichtigen. Das subjektive Wohlbefinden wurde als Mittelwertindex aus 8 Items zu psychischen, sozialen und physischen Aspekten berechnet. Der Index nimmt Werte zwischen 0 (geringstes) und 5 (höchstes) Wohlbefinden an. Aufgrund abweichender Berechnungen können keine Vergleiche mit etablierten Skalen vorgenommen werden. Anhand des nicht-parametrischen Mann-Whitney-U-Tests wurde untersucht, ob es zwischen den Befragten in SN und BW signifikante Unterschiede gibt. Dieser Fokus wurde gewählt, um einen explorativ-deskriptiven Eindruck möglicher Ost-West-Unterschiede zu erhalten.

## Ergebnisse

### Allgemeine Lebensumstände in der Coronapandemie

**Abb. 1:**
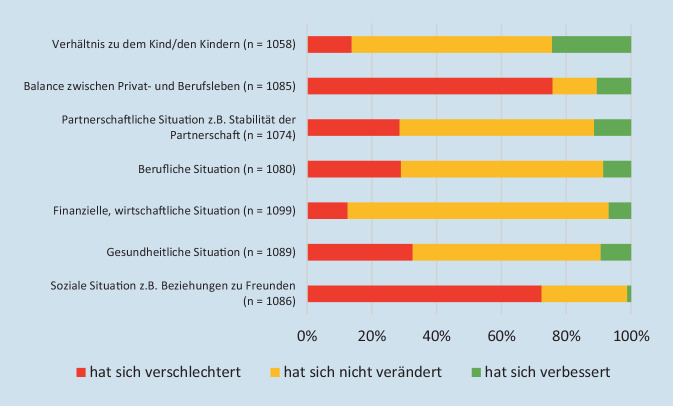
Wie hat sich Ihre Lebenssituation in der Coronapandemie im Vergleich zur Situation vorher verändert?

Die meisten Befragten geben an, dass sich ihre soziale Situation (72 %) und die Balance zwischen Privat- und Berufsleben (76 %) während der Coronapandemie verschlechtert hat. Weniger kritisch werden die finanzielle Situation oder das Verhältnis zu dem Kind/den Kindern angesehen (Abb. 1). 64 % haben *meistens* oder *die ganze Zeit* jemanden, mit dem sie über alles reden können. Insgesamt zeigen sich bei der *Beurteilung der Lebenssituation* keine signifikanten Unterschiede zwischen den Bundesländern.

Die Befragten beurteilen ihre *Gesundheit* zu 14 % als *sehr gut*, 39 % als* gut*, 26 % als *zufriedenstellend*, 18 % als *weniger gut* und 3 % als *schlecht*. Der Mittelwert der Bundesländer ist identisch bei 2,6 (*SD* = 1,0; *gut* bis *zufriedenstellend*) auf einer Skala von 1 (*sehr gut*) bis 5 (*schlecht*).

Auffällig ist hier die ungleiche Verteilung von Gesundheit zwischen den Geschlechtern: Mütter (*MD* = 2; *MW* = 2,62; *SD* = 1,01) geben einen signifikant (*U* = 126.301,50; *p* < 0,05) schlechteren Gesundheitszustand an als Väter (*MD* = 2; *MW* = 2,47, *SD* = 1,05). Auch der Index zum subjektiven *Wohlbefinden* fällt für Mütter (*MD* = 2; *MW* = 2,44; *SD* = 0,92) signifikant (*U* = 128.113,5; *p* < 0,05) geringer aus als für Väter (*MD* = 2; *MW* = 2,58; *SD* = 0,97).

Für die beiden Bundesländer zeigt der Index zum subjektiven Wohlbefinden geringe Unterschiede: in beiden Bundesländern markieren 51 % der Befragten Antwortkategorien, die auf ein mangelndes Wohlbefinden schließen lassen. Für SN ergibt sich ein Mittelwert von 2,47 (*SD* = 0,88) und für BW von 2,5 (*SD* = 0,97) aus dem Wertebereich von 0 (geringstes) bis 5 (höchstes) Wohlbefinden.

**Abb. 2:**
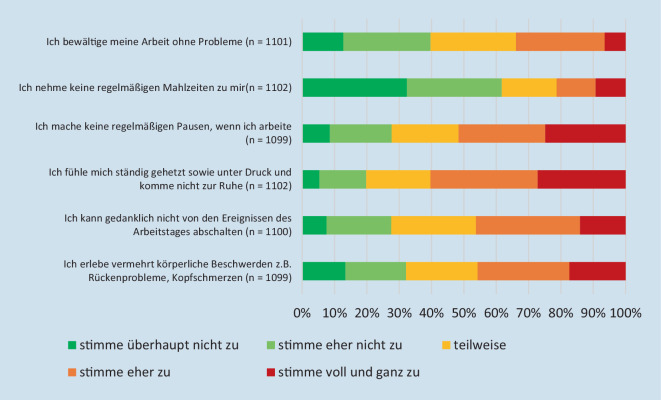
Inwieweit stimmen Sie den folgenden Aussagen in Bezug auf Ihre Berufstätigkeit in der Zeit der Coronapandemie zu oder nicht zu?

Die Ergebnisse zu den *Beanspruchungen im Arbeitskontext* zeigen, dass 60 % der Befragten *eher* bzw. *voll und ganz* zustimmen, sich ständig gehetzt sowie unter Druck zu fühlen und nicht zur Ruhe zu kommen (Abb. 2). Fast die Hälfte der Befragten erlebt vermehrt körperliche Beschwerden.

Signifikante Unterschiede zeigen, dass die Hochschulbeschäftigten in SN häufiger Probleme haben, ihre Arbeit zu bewältigen (*U* = 124.443, *p* < 0,05; SN: *n* = 373; *MD* = 3; *MW* = 2,77; *SD* = 1,131; BW : *n* = 728; *MD* = 3; *MW* = 2,94; *SD* = 1,139) und gedanklich weniger vom Arbeitstag abschalten können (*U* = 11.774; *p* < 0,01; BW: *n* = 729; *MD* = 3, *MW* = 3,16; *SD* = 1,152; SN: *n* = 371; *MD* = 4; *MW* = 3,43; *SD* = 1,13) als die Befragten in BW.

### Hochschulen in der Coronapandemie

Die Frage nach *Belastungsfaktoren in Bezug auf die Arbeitssituation* zeigt, dass die Hochschulbeschäftigten in BW bei 8 von 12 Items eine signifikant bessere Bewertung angeben als in SN. Dies äußert sich bei der Planung des Arbeitstags, der Unterstützung bei digitalen Tools, der Veränderung von Arbeitsabläufen, Verantwortungsbereichen in der Hochschule, Unterstützung durch die Führung, ergonomischer Arbeitsplatzgestaltung und beruflicher Überforderung. Der größte Belastungsfaktor in beiden Bundesländern stellt mit 75 % Zustimmung das Verschwimmen der Grenzen zwischen Arbeits- und privater Zeit dar. Hier fällt die Einschätzung in SN signifikant schlechter aus.

Die Befragten in SN empfinden ihre Arbeitszeit sowie den -aufwand als signifikant länger bzw. mehr als die Befragten in BW. Es bestehen keine signifikanten Unterschiede bei der Angst sich mit Corona anzustecken, beim sozialen Umgang sowie der fachlichen Unterstützung durch Kolleg*innen (Tab. 2).

**Tab. 2:** Belastungsfaktoren in Bezug auf die Arbeitssituation

	Bundesland	*U*-Wert	*Signifikanzwert (p)*	*Median (MD)*	*Mittelwert (MW)*	*Standardabweichung (SD)*
Durch Corona ist mein Arbeitstag schwer planbar.	BW (728)	124.294,5	0,021^a^	4	3,41	1,223
	SN (372)			4	3,6	1,148
Ich bekomme Unterstützung beim Erlernen und dem Einsatz digitaler Tools für meine Tätigkeit.	BW (662)	98.249,5	0,002^a^	3	3,25	1,221
	SN (337)			3	3,01	1,171
Ich habe Angst, mich am Arbeitsplatz mit Corona anzustecken.	BW (725)	130.159,5	0,362	2	2,21	1,249
	SN (371)			2	2,26	1,230
Seit Corona ist der Umgang mit meinen Kolleg*innen weniger freundlich und unterstützend.	BW (700)	118.453	0,165	2	1,99	1,125
	SN (356)			2	2,06	1,081
Seit Corona arbeite ich mehr und länger.	BW (722)	113.414	< 0,001^b^	3	3,03	1,310
	SN (356)			2	2,69	1,228
Seit Corona haben sich meine Arbeitsabläufe negativ verändert.	BW (722)	118.334	0,002^a^	3	3,22	1,277
	SN (369)			4	3,48	1,180
Seit Corona verschwimmen Grenzen zwischen Arbeitszeit und privater Zeit.	BW (725)	121.821	0,004^a^	4	3,99	1,156
	SN (373)			5	4,22	0,977
Seit Corona sind die Verantwortungs- und Aufgabenbereiche in der Hochschule unklar.	BW (677)	108.396	0,030^a^	2	2,28	1,143
	SN (348)			2	2,44	1,161
Seit Corona fehlt die soziale und fachliche Unterstützung durch Kolleg*innen.	BW (719)	129.961	0,573	3	3,04	1,257
	SN (369)			3	3,00	1,215
Seit Corona fehlt die Unterstützung und das Feedback durch die Führung.	BW (707)	1.144.001,5	< 0,001^b^	2	2,53	1,257
	SN (368)			3	2,80	1,281
Seit Corona hat sich die ergonomische Arbeitsplatzgestaltung verschlechtert.	BW (719)	121.073,5	0,028^a^	3	3,10	1,430
	SN (366)			4	3,30	1,425
Seit Corona fühle ich mich beruflich überfordert.	BW (721)	121.361	0,011^a^	2	2,21	1,105
	SN (370)			2	2,36	1,051

Absolute Zahlen in Klammern. MD = 1 „stimme voll und ganz zu“, 5 „stimme überhaupt nicht zu“

*SN* Sachsen, *BW* Baden-Württemberg

^*a*^*p* = signifikant

^b^*p* = höchstsignifikant

Die *Arbeitssituation unter Coronabedingungen* ist für 75 % der Befragten sinnvoll, 82 % verständlich sowie 66 % handhabbar (*stimme voll und ganz zu* und *stimme eher zu*). Der Mittelwertindex zur Resilienz aus diesen 3 Items zeigt bei den Befragten in BW signifikant (*U* = 117.990,5; *p* < 0,001) höhere Werte (*n* = 723; *MD* = 4; *MW* = 4,11; *SD* = 1,055) als in SN (*n* = 371; *MD* = 4; *MW* = 3,98; *SD* = 1,064).

Mit den *Arbeitsbedingungen zu Hause* sind 39 % aller Befragten *sehr* bzw. *eher unzufrieden*. Dabei sind Befragte in SN (*n* = 366; *MD* = 3; *MW* = 3,25; *SD* = 1,166) signifikant (*U* = 103.822,5; *p* < 0,001) unzufriedener als in BW (*n* = 700; *MD* = 3; *MW* = 2,86; *SD* = 1,130). 46 % der Befragten erleben gesundheitliche Belastungen durch die Arbeitsbedingungen zu Hause.

Auf die Frage nach *Unterstützungsangeboten durch die Hochschule* wünschen sich etwa 44 % von der Hochschulleitung, dass die Situation von Eltern mehr thematisiert wird. Danach folgt der Wunsch, zu Hause bleiben zu können, wenn ein Kind in Quarantäne muss und finanzielle Mittel für die Arbeitsplatzausstattung zu Hause.

**Abb. 3:**
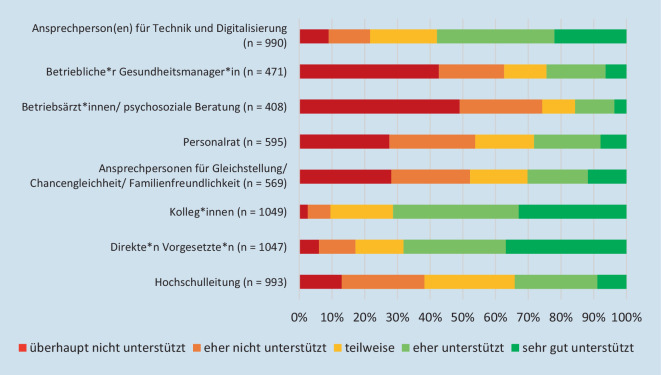
Wie bewerten Sie als Beschäftigte*r mit Kind(ern) im Kita- und Grundschulalter die Unterstützungsleistungen verschiedener Akteur*innen an Ihrer Hochschule in der Coronapandemie?

Zudem wurde nach der *Zufriedenheit der Unterstützungsleistungen verschiedener Hochschulakteure* gefragt; die Befragten in SN fühlen sich durch diese signifikant (*p* < 0,05) weniger unterstützt als in BW (Abb. 3). Die Nachfrage nach notwendigen Veränderungen von Rahmenbedingungen für Beschäftigte mit Kind(ern) im Kita- und Grundschulalter, auch nach der Coronapandemie, bejahten mehr als 80 % der Beschäftigten beider Bundesländer.

## Diskussion

Im Hinblick auf die Gesundheit und das Wohlbefinden von Hochschulbeschäftigten stellt sich insbesondere das Thema Entgrenzung als herausfordernd heraus. Politik und Hochschulen sind in der Verantwortung, Beschäftigte mit Kind(ern) bei der Vereinbarkeit von Familie und Beruf in und nach der Coronapandemie zu unterstützen und mögliche Bewältigungsstrategien umzusetzen. Mit den in der Coronapandemie umgesetzten Schutzmaßnahmen konnten Beschäftigte ihren Aufgaben als Arbeitsnehmer*in und Elternteil oft nicht gleichermaßen gerecht werden [5]. Zwar belegen frühere Studien und Metanalysen zu den Folgen von flexiblen Arbeitszeitmodellen, wie bspw. Homeoffice, verschiedene positive Effekte auf die Lebensqualität und die Vereinbarkeit von Familie und Beruf [7, 22], doch zu vermuten ist, dass es durch die zusätzlichen Herausforderungen der Pandemie wie Homeschooling und soziale Isolation zu einer Kumulation von Belastungen kommen kann, die kritisch zu reflektieren sind. Dies deuten die Befunde zu den Geschlechtsunterschieden an. Weitere Studienergebnisse sprechen ebenfalls dafür, dass Mütter während den Kita- und Schulschließungen überwiegend die Kinderbetreuung übernommen und damit die Hauptlast getragen haben. Bereits zuvor bestehende geschlechterspezifische Rollen- und Arbeitsverteilungen von Müttern und Vätern wurden dadurch verstärkt [11]. Das mehrdimensionale Konstrukt des Wohlbefindens sowie die subjektive Gesundheit bewerten die befragten Mütter in der vorliegenden Studie deutlich kritischer als die Väter.

Die Befunde zeigen, dass es wichtig ist, eine hohe Transparenz von Coronainformationen für alle Hochschulmitglieder zu gewährleisten und insbesondere Hochschulleitungen für die Situation von Beschäftigten mit Kind(ern) im Kita- und Grundschulalter zu sensibilisieren. Zu ihrem Aufgabenbereich gehört die Durchführung regelmäßiger psychischer Gefährdungsbeurteilungen, um frühzeitig notwendige Interventionen einzuleiten [5]. Dies sollte auch in Ausnahmesituationen erfolgen.

Die durchgeführte Studie zeigt, dass die Coronapandemie negative Auswirkungen auf das Wohlbefinden der Hochschulbeschäftigten mit Kind(ern) in SN und BW hat. Die Befragten in BW bewerten ihre Arbeitssituation sowohl in der Hochschule als auch zu Hause in vielen Aspekten positiver als in SN. Möglicherweise werden die unterschiedlichen Bewertungen durch unterschiedliche Strukturen, Ressourcen und Erfahrungen im Bereich des betrieblichen Gesundheitsmanagements oder coronabedingt durch die jeweilige Inzidenzlage sowie die politischen Regelungen innerhalb der Bundesländer beeinflusst. In der zweiten Coronawelle wies SN ab November 2020 eine durchgehend höhere Inzidenzrate auf als BW [18]. Der Anteil der befristet Beschäftigten in unserer Studie ist in SN um etwa 16 Prozentpunkte größer als in BW. Die höhere Befristungsquote könnte zu dem höheren Belastungsempfinden in SN führen, da die Beschäftigten ihre Arbeitsplatzsicherheit eventuell durch ein entsprechendes berufliches Engagement gewährleisten wollen. Zudem bestand für Hochschulbeschäftigte in SN bei fehlender Systemrelevanz beider Elternteile kein Anspruch auf eine Notbetreuung [19]. In BW wurde dieser Anspruch im zweiten Lockdown wesentlich niederschwelliger ermöglicht [21]. Generell arbeiten Mütter in Ostdeutschland mehr in Vollzeitstellen als in Westdeutschland [23], was auch die vorliegende Studie bestätigt. Dies könnte sich auch auf das Belastungsempfinden in der Krise auswirken. Diese Unterschiede stellten Hochschulbeschäftigte mit Kind(ern) in SN wohl vor größere Herausforderungen bei der Vereinbarkeit von Arbeit und Kinderbetreuung als in BW. Weitere Analysen nach Geschlecht mit einem besonderen Fokus auf die vulnerable Gruppe von befristet beschäftigten Wissenschaftlerinnen mit Kind(ern) sind vorgesehen und sollen die Ergebnisse vervollständigen.

## Limitierung der Studie

- Keine Repräsentativität für Hochschulbeschäftigte in BW und SN,
- keine Kontrollierbarkeit bei der Verteilung des Fragebogen-Links aufgrund des Verteilungsverfahrens durch verschiedene Praxispartner*innen,
- keine Vergleichs- und Verlaufsdaten von Hochschulbeschäftigten in Bezug auf Gesundheit und Wohlbefinden vor der Coronapandemie.

## Fazit für die Praxis

- Die negativen sowie positiven Auswirkungen von Homeoffice/Telearbeit und damit einhergehende Beanspruchungen müssen Beachtung im betrieblichen Gesundheitsmanagement von Hochschulen finden und in die psychische Gefährdungsbeurteilung aufgenommen werden, auch in Ausnahmesituationen.
- Die Gefährdungsbeurteilung gehört zum Aufgabebereich der Hochschulleitung. Weitere Akteure wie Betriebsärzt*innen, Fachkräfte für Arbeitssicherheit, Personalvertretung, Betriebliches Gesundheitsmanagement und Gleichstellung sollten einbezogen werden.
- Hochschulbeschäftigte mit Kind(ern) sollten beteiligt werden, um Bedarfe offenzulegen und zielgruppengerechte Maßnahmen, besonders beim Thema Entgrenzung, zu entwickeln.
- Wichtig ist eine offene und wertschätzende Kommunikation der Hochschulleitung für die Situation von Beschäftigten mit Kind(ern) auch nach der Pandemie.
